# Antihypertensive effects of rosuvastatin in patients with hypertension and dyslipidemia: A systemic review and meta-analysis of randomized studies

**DOI:** 10.1371/journal.pone.0260391

**Published:** 2021-11-24

**Authors:** Sungjae Lee, Seungwon Yang, Min Jung Chang

**Affiliations:** 1 Department of Pharmaceutical Medicine and Regulatory Sciences, Colleges of Medicine and Pharmacy, Yonsei University, Incheon, Republic of Korea; 2 Department of Pharmacy and Yonsei Institute of Pharmaceutical Science, College of Pharmacy, Yonsei University, Incheon, Republic of Korea; 3 Department of Industrial Pharmaceutical Science, Yonsei University, Incheon, Republic of Korea; Universidade Federal da Bahia, BRAZIL

## Abstract

Some studies have suggested the antihypertensive effects of statins, a class of lipid-lowering agents, particularly in patients with hypertension. However, the evidence for the role of statins in blood pressure (BP) lowering is controversial, and no meta-analysis of rosuvastatin therapy has been conducted to assess its BP-lowering effects. Therefore, the aim of this meta-analysis of randomized controlled trials (RCTs) was to investigate the effects of rosuvastatin on systolic blood pressure (SBP) and diastolic blood pressure (DBP) in patients with hypertension. We systematically searched the electronic databases MEDLINE, EMBASE, and Cochrane Library to identify RCTs in which patients were assigned to groups of rosuvastatin plus antihypertensive agents vs. antihypertensive agents. The three authors independently selected the studies, extracted data, and assessed methodological quality. We included five RCTs in this meta-analysis with 288 patients treated with rosuvastatin and 219 patients without rosuvastatin. The mean DBP in the rosuvastatin group was significantly lower than that in the non-rosuvastatin group by −2.12 mmHg (95% confidence interval (CI) −3.72 to −0.52; *P*_fixed-effects model_ = 0.009; *I*^*2*^ = 0%, *P*_heterogeneity_ = 0.97). Rosuvastatin treatment also lowered the mean SBP compared with the non-rosuvastatin treatment by −2.27 mmHg, but not significantly (95% CI − 4.75 to 0.25; *P*_fixed-effects model_ = 0.08; *I*^*2*^ = 0%, *P*_heterogeneity_ = 0.82). In this study, we reviewed the antihypertensive effects of rosuvastatin in patients with hypertension and dyslipidemia. We demonstrated a modest significant reduction of DBP and a trend toward a lowered SBP in patients with hypertension with rosuvastatin therapy. Rosuvastatin could be beneficial to control hypertension and, consequently, contribute toward reducing the risk of cardiovascular events in patients with hypertension and dyslipidemia.

## Introduction

Cardiovascular disease (CVD), the leading cause of death globally, accounted for more than 17.6 million deaths in 2016 [1]. Of many risk factors, hyperlipidemia, with elevated lipoprotein levels, and arterial hypertension, with elevated systolic blood pressure (SBP) and diastolic blood pressure (DBP), are among the major risk factors that are independently or positively associated with the development of CVD, including myocardial infarction, stroke, and congestive heart failure [2, 3]. In a large proportion of patients, hyperlipidemia coexists with hypertension [4–6]. Furthermore, patients who have concomitant hyperlipidemia and hypertension are at a higher risk of CVD than patients who have hyperlipidemia or hypertension alone. Therefore, the combination therapy of antihypertensive agents with statins has been used for the control of both BP and the blood cholesterol level [7].

Recently, studies have demonstrated that statins, a class of lipid-lowering agents that inhibit 3-hydroxy-3-methylglutaryl coenzyme A reductase, are associated with an unexpected reduction in BP in patients with hypertension [8–10]. The underlying mechanism of the BP-lowering effect of statins could be related to the modulation of endothelial function and vascular oxidative stress [9]. A reduction in BP conferred by statin treatment could provide additional cardiovascular protection in patients with hypertension [10]. However, studies on the effects of statins on BP have shown conflicting results, possibly owing to the heterogeneity of the antihypertensive agents, different initial BP levels, and different age groups used [11–13]. Additionally, although rosuvastatin is one of the most potent and commonly prescribed statins for the treatment of hyperlipidemia, the effects of rosuvastatin have not been analyzed in previous meta-analyses that report the antihypertensive effects of statins [14].

Therefore, this meta-analysis was conducted to investigate whether rosuvastatin can lower DBP and SBP in patients with hypertension and dyslipidemia.

## Materials and methods

We followed the Preferred Reporting Items for Systematic reviews and Meta-analyses (PRISMA) guidelines to report the study design, search strategy, data analysis, and results of this meta-analysis (S1 Appendix). Using a standardized pilot-tested form, two authors (SL and MJC) independently checked the fulfillment of study eligibility, assessed the risk of bias, and conducted the extraction of data from each eligible study. Disagreements were resolved by consensus in the presence of a third author (SL, SY, and MJC).

### Study selection

We included randomized controlled trials (RCTs) that investigated the effect of rosuvastatin on BP. Studies were eligible if they met the following criteria: (1) participants: patients with hypertension; (2) intervention: rosuvastatin treatment; (3) comparison: standard care or placebo; (4) outcome: changes in DBP or SBP measurements from the baseline to the end-of-treatment period. We excluded studies where we were unable to calculate the mean difference of the DBP and SBP measurements between the baseline and the end-of-treatment period in the intervention and control groups. We also excluded studies that did not provide any of the outcomes of interests: the number of patients in the intervention and control groups and the description of the demographic characteristics of the study population.

### Search strategy

We systematically searched the electronic databases MEDLINE, EMBASE, and the Cochrane Library for Central Register of Clinical Trials from earliest available to February 2020, using the following keywords and MeSH terms: “hypertension,” “blood pressure,” and “antihypertensive effects.” The detailed search strategies are provided in S2 Appendix. We restricted our search to clinical trials, RCTs, and English-language articles. No posters or abstracts were included. The reference lists of the screened articles and related systematic reviews and meta-analyses were manually reviewed for additional relevant studies.

### Data collection and quality assessment

We used a standardized data extraction form to collect the following information from each eligible study: study characteristics (author, publication year, study design and study phase, and number of study sites), participant characteristics (sex, age, and diagnosed disease), details of treatment regimens in the intervention and control groups (run-in period, drug name, dose, and duration), and outcome measures (DBP and SBP measurements at the baseline and changes in the DBP and SBP from the baseline to the end-of-treatment period).

We used a modified version of the Cochrane Collaboration’s Risk of Bias tool [15] for the quality assessment according to the following categories: 1) sequence generation; 2) allocation concealment; 3) blinding of participants and outcome assessors; 4) incomplete outcome data; and 5) selective outcome reporting. We then judged whether each included trial was at a high, low, or unclear risk of bias.

### Data analysis

The primary outcome was a change in the DBP and SBP measurements from the baseline to the end-of-treatment period and the difference in BP between the intervention and control groups. The continuous data are summarized as mean differences (MDs) and standard deviations (SDs). In cases in which the SD could not be obtained from a selected study, we calculated the SD using the “Finding Standard Deviation” tool provided in the Cochrane Handbook [16]. We calculated the overall summary estimates and 95% confidence intervals (CIs) using the inverse variance fixed-effects model because heterogeneity using the random-effects model was low. Heterogeneity was assessed using a Q^2^ test and the *I*^2^ statistic with significance at *P* < 0.1 [17].

We conducted a subgroup analysis based on treatment regimen (dual therapy with rosuvastatin and one antihypertensive agent vs. triple therapy with rosuvastatin and two antihypertensive agents). Furthermore, publication bias was assessed visually via the asymmetrical shape of the funnel plot. Statistical analyses were performed using Review Manager Software version 5.3 (Cochrane Collaboration, London, UK).

## Results

### Study selection

**Fig 1:**
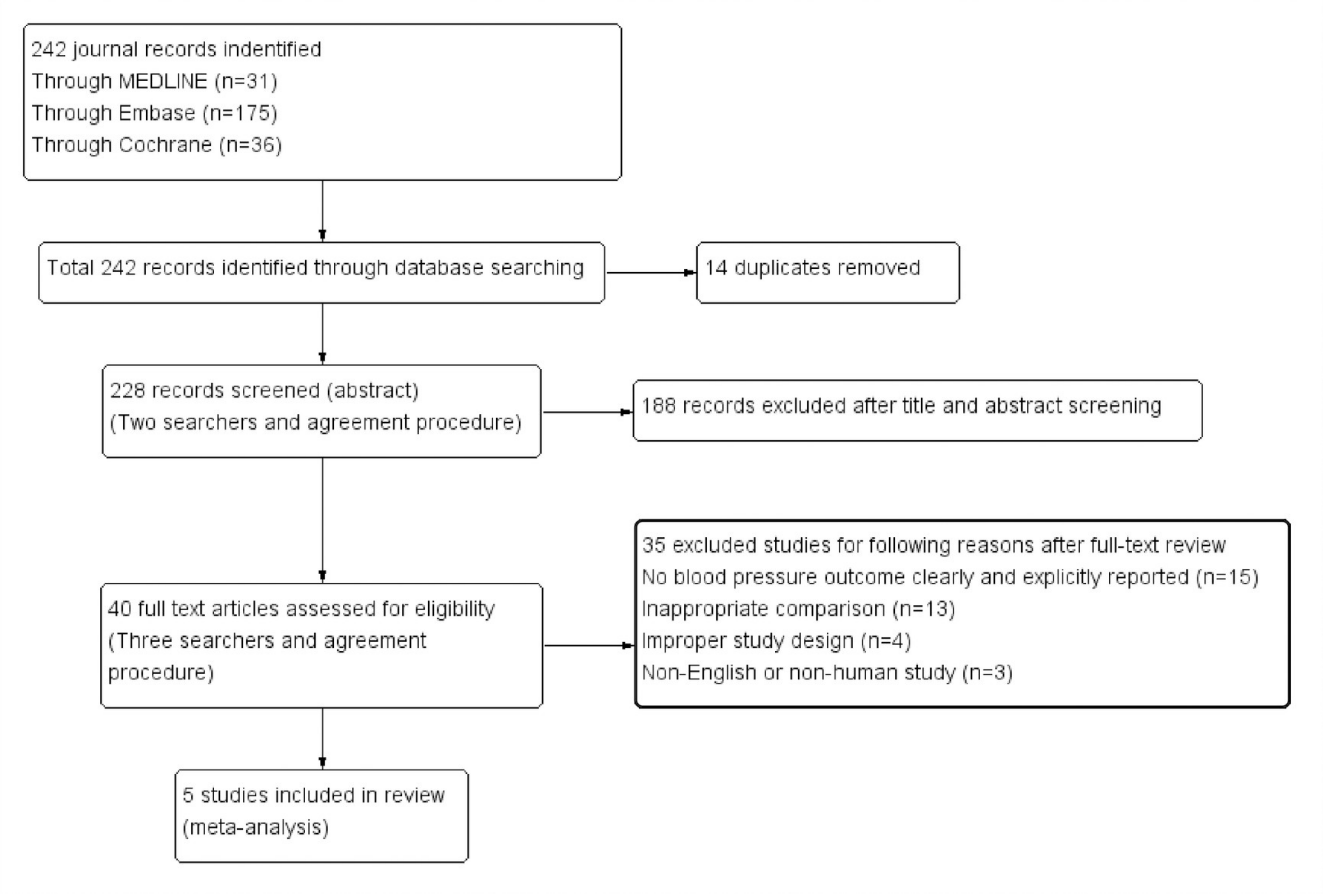
Preferred Reporting Items for Systematic Reviews and Meta-Analyses (PRISMA) flow diagram of study selection.

In total, 242 studies were identified through database searching. After removing 14 duplicates, we screened 228 titles and abstracts for study relevance and discarded 188 studies. Then, 40 full-text studies were carefully reviewed for study eligibility, and 35 studies were removed. Finally, a total of five studies that met the inclusion criteria were included in this meta-analysis (Fig 1).

### Baseline characteristics

This meta-analysis included 507 adult patients with hypertension and dyslipidemia: 288 patients were assigned to rosuvastatin 20 mg/day and 219 patients to placebo or standard care. The included studies were multicenter, phase III trials conducted in South Korea. The mean age of the patients in the intervention group was similar to that of those in the control group (61.85 years vs. 62.07 years, respectively). The majority of the participants were male. Participants in the intervention group received combination therapy comprising rosuvastatin and angiotensin II receptor blockers (ARBs; fimasartan, telmisartan, or olmesartan) in three studies and a triple combination comprising rosuvastatin/calcium channel blockers/ARBs (rosuvastatin/amlodipine/losartan or rosuvastatin/amlodipine/telmisartan) in two studies. Participants in the control group received placebo or standard care. The duration of treatment was 8 weeks in all studies. In the intervention group, the baseline mean SBP ranged from 142.74 mmHg to 152.5 mmHg and the baseline mean DBP ranged from 81.74 mmHg to 94.24 mmHg. Overall, the baseline characteristics of the patients randomized to the intervention and control groups were similar (Table 1).

**Table 1:** Characteristics of the studies included in the meta-analysis (n = 5).

Author (year)	Study design & phase	Study sites, n	Country	Subjects, n		Male, %	Mean age, years		Disease diagnosed	Medications administered, mg/d		Duration, weeks	Baseline mean SBP/DBP, mmHg^1^	
				Intervention	Control		Intervention (SD)	Control (SD)		Intervention	Control		Intervention (SD)	Control (SD)
Lee et al (2017) [18]	Multicenter, Randomized, double-blind, placebo-controlled, phase III	23	South Korea	54	46	74.8	60.3 (8.4)	59.8 (9.0)	Essential hypertension and hyperlipidemia	Losartan 100 + amlodipine 5 + rosuvastatin 20	Losartan 100 + amlodipine 5	8	142.74 (13.64)/ 94.24 (6.95)	143.54 (14.39)/ 95.18 (7.14)
Rhee et al (2017) [19]	Multicenter, randomized, double-blind, parallel, phase III	29	South Korea	46	45	73.3	59.3 (8.7)	62.3 (9.5)	Hypertension and dyslipidemia	Fimasartan 120 + rosuvastatin 20	Fimasartan 120	8	152.5 (9.9)/ 89.4 (8.3)	151.3 (9.0/ 85.8 (9.3)
Oh et al (2018) [20]	Multicenter, randomized, 4-arm, double-blind, placebo-controlled, phase III	21	South Korea	80	43	73.9	59.8 (10.9)	62.1 (10.9)	Hypertension and dyslipidemia	FDC of telmisartan 80 /rosuvastatin 20	Telmisartan 80	8	151.8 (11.4)/ 91.2 (10.1)	149.9 (14.7/ 89.5 (10.0)
Park et al (2016) [21]	Randomized, double-blind, factorial-design, phase III	25	South Korea	61	36	64.2	61.9 (8.1)	59.5 (6.9)	Hypertension and dyslipidemia	FDC of olmesartan 40/ rosuvastatin 20	Olmesartan 40	8	150.6 (11.9)/ 92.0 (7.4)	150.6 (15.5/ 93.3 (5.0)
Hong et al (2019) [22]	Multicenter, randomized, double-blind, parallel, phase III	9	South Korea	47	49	68.0	67.9 (9.4)	66.6 (10.2)	Hypertension and dyslipidemia	Two FDC tablets of telmisartan 40/ amlodipine 5 + one tablet of rosuvastatin 20	Two FDC tablets of telmisartan 40/ amlodipine 5	8	149.49 (12.09)/ 81.74 (12.52)	144.29 (11.09)/ 77.84 (12.78)

^1^ Sitting systolic blood pressure and sitting diastolic blood pressure

Abbreviations: FDC, fixed-dose combination; SBP, systolic blood pressure; DBP, diastolic blood pressure; SD, standard deviation

### Effect of rosuvastatin on DBP and SBP

**Fig 2:**
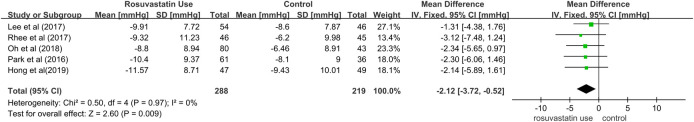
Pooled mean differences for the outcomes of diastolic blood pressure changes in patients with hypertension receiving rosuvastatin therapy vs. placebo or standard care.

**Fig 3:**
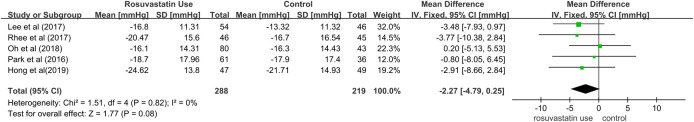
Pooled mean differences for the outcomes of changes in systolic blood pressure in patients with hypertension receiving rosuvastatin therapy vs. placebo or standard care.

We generated a pooled estimate for the MD of BP from the baseline to the end-of-treatment period for the rosuvastatin group vs. control group. The patients treated with rosuvastatin in addition to antihypertensives had a significantly lower DBP (MD = −2.12 mmHg; 95% CI = −3.72 to −0.52; *P*_fixed-effects model_ = 0.009; *I*^*2*^ = 0%, *P*_heterogeneity_ = 0.97) than the patients treated with antihypertensives alone (Fig 2). However, rosuvastatin therapy was not associated with a significant reduction in SBP (MD = −2.27 mmHg; 95% CI = −4.79 to 0.25; *P*_fixed-effects model_ = 0.08; *I*^*2*^ = 0%, *P*_heterogeneity_ = 0.82) (Fig 3).

**Fig 4:**
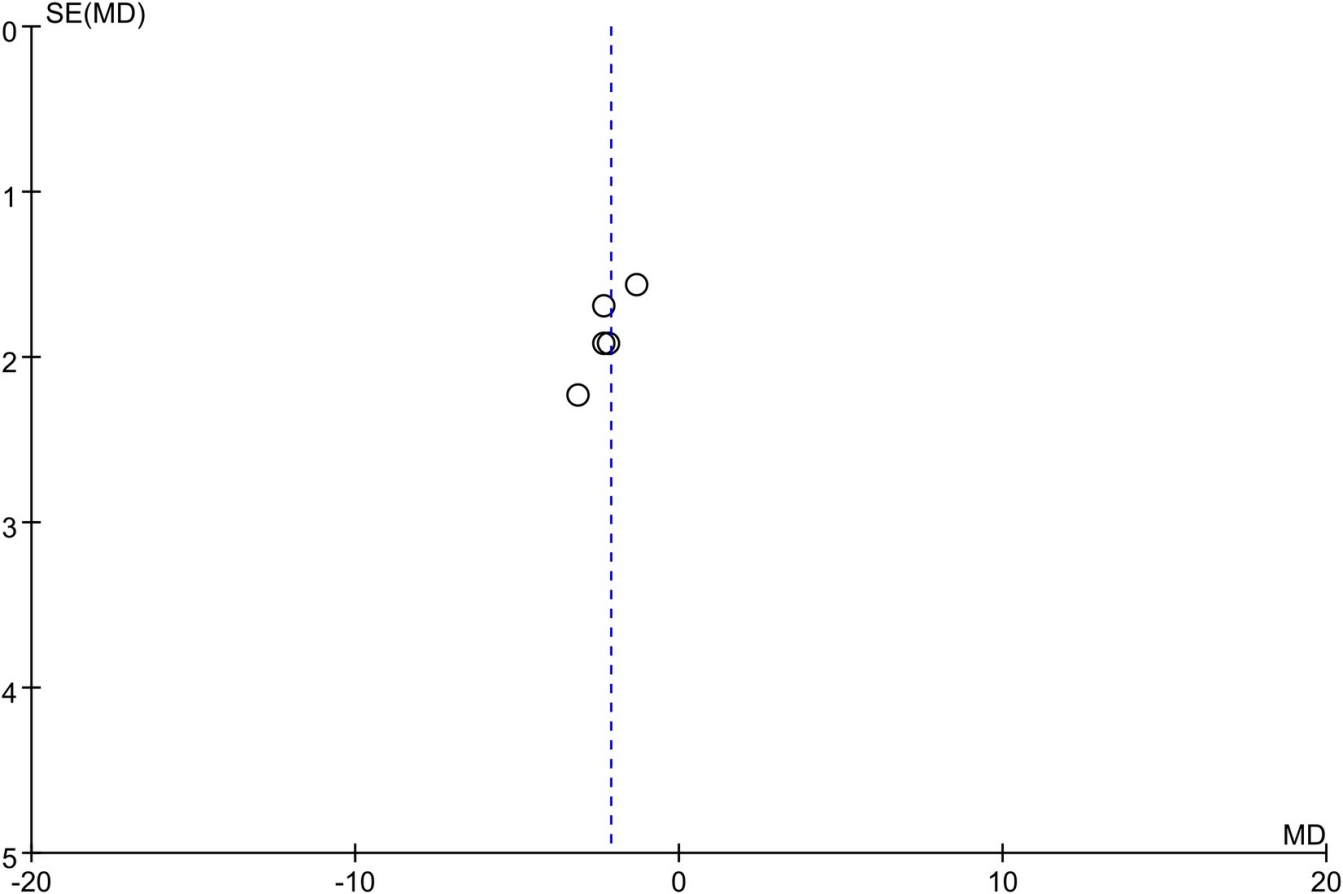
Funnel plot for outcomes of changes in diastolic blood pressure.

**Fig 5:**
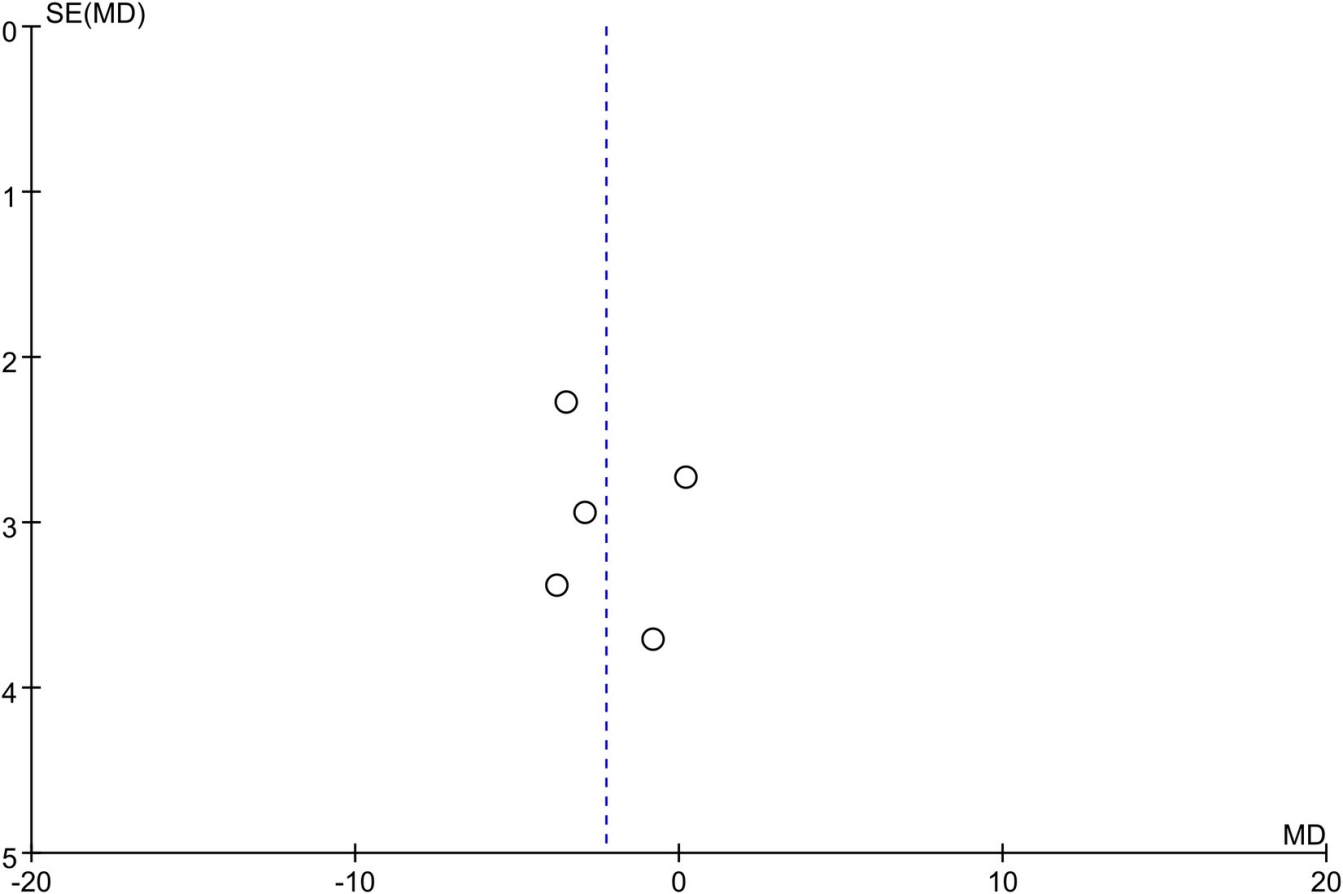
Funnel plot for outcomes of changes in systolic blood pressure.

The funnel plot for the outcomes of changes in the DBP and SBP showed symmetry, which suggested a low risk of publication bias (Figs 4 and 5).

### Subgroup analysis

**Fig 6:**
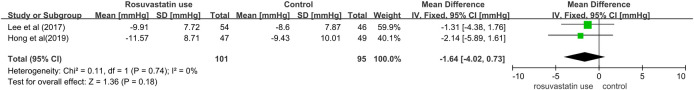
Pooled mean differences for the outcomes of diastolic blood pressure changes in patients with hypertension treated with a combination of rosuvastatin and one antihypertensive agent.

**Fig 7:**
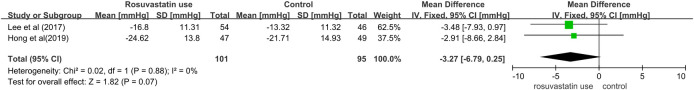
Pooled mean differences for the outcomes of systolic blood pressure changes in patients with hypertension treated with a combination of rosuvastatin and one antihypertensive agent.

We conducted a subgroup analysis on the MD of BP outcomes according to therapy regimens: dual combination (one antihypertensive agent + rosuvastatin) and triple combination (two antihypertensive agents + rosuvastatin). In two trials, the enrolled patients treated with a dual combination (one antihypertensive agent + rosuvastatin) did not have a significantly lower DBP (MD = −1.64 mmHg; 95% CI = −4.02 to 0.73; *P*_fixed-effects model_ = 0.18; *I*^*2*^ = 0%, *P*_heterogeneity_ = 0.74) or SBP (MD = −3.27 mmHg; 95% CI = −6.79 to 0.25; *P*_fixed-effects model_ = 0.07; *I*^*2*^ = 0%, *P*_heterogeneity_ = 0.88) than the patients in the control group (Figs 6 and 7).

**Fig 8:**
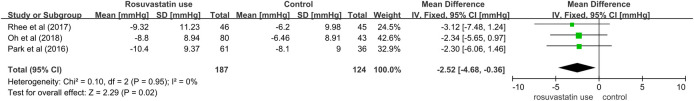
Pooled mean differences for the outcomes of diastolic blood pressure changes in patients with hypertension treated with a triple combination of rosuvastatin plus two antihypertensive agents.

**Fig 9:**
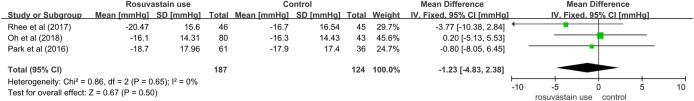
Pooled mean differences for the outcomes of systolic blood pressure changes in patients with hypertension treated with a triple combination of rosuvastatin plus two antihypertensive agents.

In three studies, the patients who received a triple combination of rosuvastatin plus two antihypertensive agents showed a lower DBP (MD = −2.52 mmHg; 95% CI = −4.68 to −0.36; *P*_fixed-effects model_ = 0.02; *I*^*2*^ = 0%, *P*_heterogeneity_ = 0.95) than the patients who received two antihypertensives (Fig 8). Although the triple combination showed a trend toward a lower SBP than the control treatment, it was not statistically significant (MD = −1.23 mmHg; 95% CI = −4.83 to 2.38; *P*_fixed-effects model_ = 0.50; *I*^*2*^ = 0%, *P*_heterogeneity_ = 0.65) (Fig 9).

### Quality assessment within studies (risk of bias)

**Fig 10:**
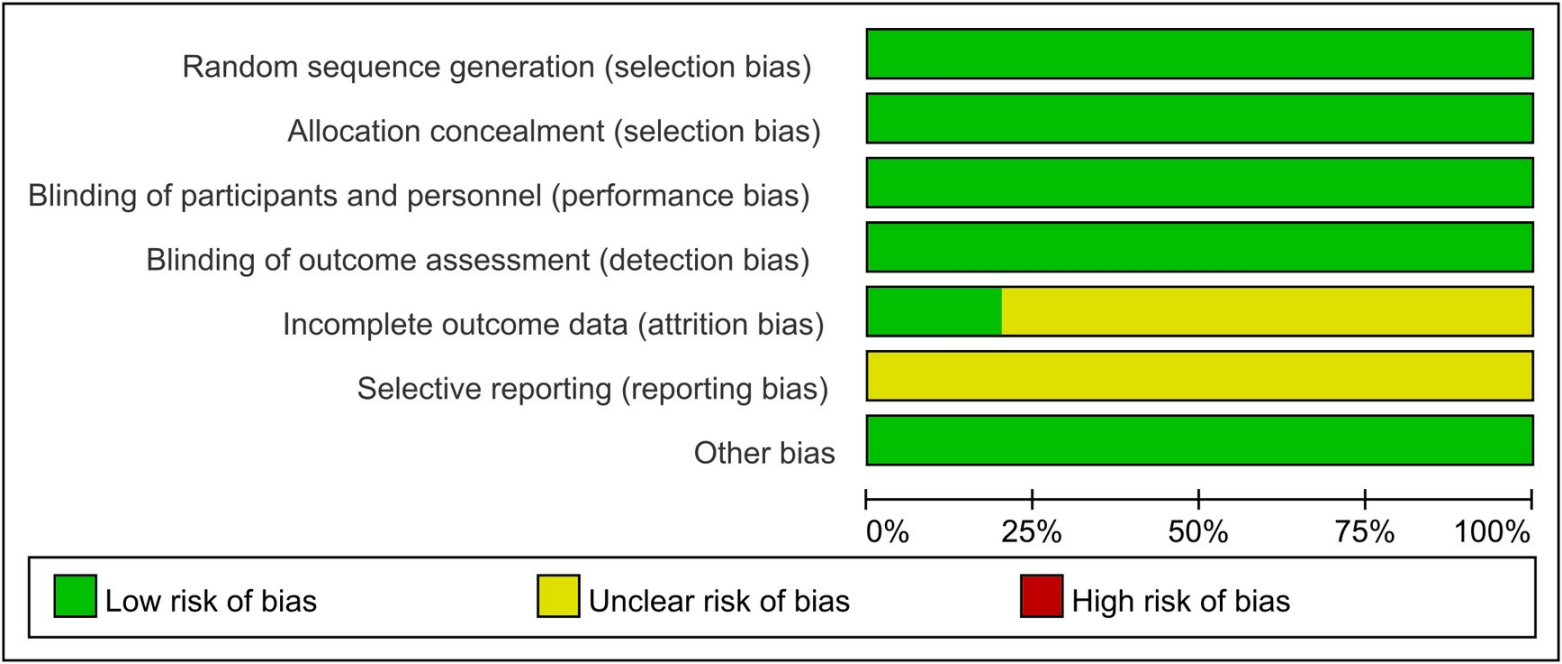
Risk of bias.

Fig 10 shows the results of the risk of bias assessment. All studies were assessed as being of low risk of bias for the following aspects: selection, performance, detection, and others. Four studies [18–20, 22] were assessed as being of unclear risk of bias for attrition because the reasons that patients were lost to follow-up and stopped treatment were not described. All studies included in the analysis were assessed as being of unclear risk of bias for reporting because the study protocols were not available and it was unclear whether all pre-specified and expected outcomes of interest were reported.

## Discussion

In this meta-analysis of RCTs, we investigated the BP-lowering effects of rosuvastatin in patients with hypertension and dyslipidemia. A key insight from our analysis is that rosuvastatin 20 mg in addition to antihypertensive agents significantly reduced the DBP in patients with hypertension. There was also a trend toward a lower SBP in patients who received rosuvastatin therapy than in those who did not (−2.12 mmHg in DBP and −2.27 mmHg in SBP).

Kanukula et al. presented that previous systematic reviews reported conflicting results about the BP-lowering effect of statins and there was a difference in change in DBP associated with antihypertensive drugs + rosuvastatin versus antihypertensive drugs alone (−3.1 mm Hg; 95% CI = −5.2 to −0.9) [23]. To our knowledge, this is the first meta-analysis to evaluate the BP-lowering effect of rosuvastatin. This meta-analysis provides updated, compelling evidence on the expanding role of rosuvastatin in BP reduction.

Rosuvastatin is in a class of lipid-lowering medication which is inhibition of 3-hydroxy-3-methyl-glutaryl coenzyme a (HMG-CoA) reductase called statins [24]. The U.S. Food and Drug Administration (FDA) approved the rosuvastatin for the treatment of high triglycerides in adults, for patients with primary dysbetalipoproteinemia, and patients with homozygous familial hypercholesterolemia [25]. Thus, there is a lack of evidence to support BP-lowering effect of rosuvastatin with regard to the mechanism of action and indications.

However, the results of this study demonstrated the beneficial effects of rosuvastatin therapy on DBP, which is consistent with those of previous studies [26, 27]. Heo *et al*. reported that the mean change in DBP with rosuvastatin monotherapy is significantly different (each study varied in −1.4 ~ −2.8 mmHg, *P* < 0.05) than without and that rosuvastatin may produce a modest antihypertensive effect in DBP [26]. Another study, by Jang *et al*., involved an RCT to evaluate the additive beneficial effects of rosuvastatin combined with valsartan. The results showed that combined therapy with rosuvastatin and valsartan has a significant DBP-lowering effect compared with valsartan monotherapy; however, this effect was not observed for SBP (−3.9 mmHg for DBP, *P* = 0.02; and −2.4 mmHg in SBP, *P* = 0.42) [27].

The BP-lowering effect of statins may be the result of a pleiotropic effect [28], which may enhance the reversal of vascular endothelial dysfunction [29]. There has been an increase in the number of both *in vitro* and animal studies showing that statins exert pleiotropic effects such as increased bioavailability of nitric oxide, increased endothelial-dependent vasodilation, and reduced levels of endothelin-1, which lead to a reduction in cardiovascular risk [30–32]. This phenomenon may be clinically important in patients with hypertension and dyslipidemia because a reduction in cardiovascular risk can eventually lead to a reduction in morbidity and mortality [26].

This meta-analysis demonstrated the possibility of reducing SBP in patients with hypertension who receive rosuvastatin therapy, which is consistent with the results of a previous study [13]. A meta-analysis by Strazzullo *et al*. showed a significant reduction in SBP, but a trend of reduction in DBP, with statin therapy (−1.2 mmHg for DBP, *P* = 0.023; and −4.0 mmHg for SBP, *P* = 0.066) in patients who have a baseline DBP ≥ 80 mmHg or SBP ≥ 130 mmHg [13]. However, another meta-analysis of prospective-controlled studies evaluated the antihypertensive effects of statins and found a small but significant reduction in both DBP and SBP (−0.94 mmHg for DBP and −2.62 mmHg for SBP) in patients with diabetes who use statins [33]. Additionally, two studies reported that statin use is related to both a DBP- and SBP-lowering effect when co-administered with antihypertensive agents [12, 34].

The results of our subgroup analyses showed a difference in BP reduction by therapy regimen: dual combination (one antihypertensive agent + rosuvastatin) and triple combination (two antihypertensive agents + rosuvastatin). Triple combination therapy with rosuvastatin and two antihypertensive agents showed a significant reduction in DBP only (−2.52 mmHg for DBP, *P*_fixed-effects model_ = 0.02).

Discrepancies between the results of different studies may be explained by the heterogeneity of the study groups (e.g., patient populations with various diagnoses, different ages of patients, initial levels of BP and cholesterol, or administration of antihypertensive drugs) and methodological differences (e.g., various techniques of BP measurement) [35].

Our results demonstrated an average decrease of 2.12 mmHg in DBP and an average decrease of 2.27 mmHg in SBP with rosuvastatin use, which is a reduction in BP similar to that observed in other studies that evaluated the BP-lowering effects of statins [10, 13, 33]. BP control may be of paramount importance to decrease CVD risk. For example, some studies have reported that small reductions in BP (1–2 mmHg) markedly decrease the morbidity and mortality from CVD [36, 37] and a reduction in SBP by 3–5 mmHg reduces stroke risk by 2–3% [38]. The Systolic Blood Pressure Intervention Trial reported the benefits of lowering the BP to 120 mmHg in some high-risk groups of patients [39]. Therefore, the additive BP-lowering effect of rosuvastatin observed in this meta-analysis may decrease CVD risk in patients with hypertension.

However, this study had several limitations. First, all studies included in this meta-analysis were conducted in Korea, which limits the generalizability of this study to other ethnic groups. Second, only 20 mg/day of rosuvastatin was included in this analysis; thus, the BP response at different doses of rosuvastatin was not addressed. However, this is because, owing to regulations, clinical trials must test the efficacy and safety at the highest doses of each component. Third, the studies included in this meta-analysis varied in several aspects, including the inclusion and exclusion criteria, study design, and use of concomitant medications. Finally, as in other meta-analyses, given the lack of data in each trial, compliance with the assigned therapy could not be considered in this analysis.

## Conclusions

In conclusion, our results suggest that rosuvastatin exerts a modest DBP-lowering effect in patients with hypertension and dyslipidemia when combined with antihypertensive agents. This study adds to our understanding of the BP-lowering effects of statins, which are currently the best-selling prescription drugs worldwide. Further studies are required to evaluate whether the BP-lowering effects of rosuvastatin therapy can be translated into clinical practice.

## Supporting information

S1 Appendix PRISMA checklist. (DOC)

S2 Appendix Search strategies. (DOCX)
