# Supplementary material for: Antihypertensive effects of rosuvastatin in patients with hypertension and dyslipidemia: A systemic review and meta-analysis of randomized studies
Source: PLoS One. 2021 Nov 24;16(11):e0260391. doi: 10.1371/journal.pone.0260391 (PMC8612562; doi:10.1371/journal.pone.0260391)
Supplement: S2 Appendix — (DOCX) [file pone.0260391.s002.docx]

**Appendix 2. Search Strategies [posted as supplied by author]**

**The following databases were searched:**

- **Medline**
- **EMBASE**
- **Cochrane Library**

| **Databases** | **No,** | **Search term** | **Results** |
| --- | --- | --- | --- |
| **Medline** | **1** | **(“dyslipidemia”[Mesh] OR “hypertension”[Mesh] OR “dyslipidemia”[tiab] OR “hypertension”[tiab])** | **481984** |
|  | **2** | **("rosuvastatin"[tiab] OR "rosuvastatin"[Mesh])** | **2994** |
|  | **3** | **("antihypertensive"[tiab] OR ("antihypertensive"[Mesh] OR "hypertensi*"[Mesh] OR "hypertensi*"[tiab] OR "blood pressure"[Mesh] OR "blood pressure"[tiab])** | **600116** |
|  | **4** | **("antihypertensive"[tiab] OR "hypertensi*"[tiab])** | **348437** |
|  | **5** | **(random*[tw] OR "controlled trial"[tiab] OR “clinical trial”[tiab] OR "trial"[tiab])** | **1446898** |
|  | **6** | **1 AND 2 AND 3 AND 4 AND 5** | **31** |
| **EMBASE** | **1** | **('hypertension'/exp OR 'blood pressure'/exp OR 'hyperlipidemia'/exp OR 'dyslipidemia'/exp)** | **1203347** |
|  | **2** | **('rosuvastatin'/exp)** | **13791** |
|  | **3** | **('antihypertensive':ti,ab)** | **134670** |
|  | **4** | **('clinical trial'/all OR 'controlled study'/exp OR 'randomized controlled trial'/exp)** | **7355411** |
|  | **5** | **1 AND 2 AND 3 AND 4** | **175** |
| **Cochrane Library** | **1** | **dyslipidemias** | **6731** |
|  | **2** | **rosuvastatin** | **2259** |
|  | **3** | **antihypertens* or hypertens* or blood pressure or pressure** | **164288** |
|  | **4** | **controlled trial or clinical trial or random* or trial** | **1210277** |
|  | **5** | **english** | **939311** |
|  | **6** | **1 AND 2 AND 3 AND 4 AND 5** | **36** |
